# Plant-derived bioactive compounds regulate the NLRP3 inflammasome to treat NAFLD

**DOI:** 10.3389/fphar.2022.896899

**Published:** 2022-08-09

**Authors:** Qian Huang, Xin Xin, QinMei Sun, Ziming An, Xiaojun Gou, Qin Feng

**Affiliations:** ^1^ Institute of Liver Diseases, Shuguang Hospital Affiliated to Shanghai University of Traditional Chinese Medicine, Shanghai, China; ^2^ Central Laboratory, Baoshan District Hospital of Integrated Traditional Chinese and Western Medicine of Shanghai, Shanghai, China; ^3^ Shanghai Key Laboratory of Traditional Chinese Clinical Medicine, Shanghai, China; ^4^ Key Laboratory of Liver and Kidney Diseases, Shanghai University of Traditional Chinese Medicine, Ministry of Education, Shanghai, China

**Keywords:** non-alcoholic fatty liver disease, NLRP3 inflammasome, plant-derived active ingredients, terpenoids, flavonoids, alkaloids, phenols

## Abstract

Non-alcoholic fatty liver disease (NAFLD) is a liver disorder characterized by abnormal accumulation of hepatic fat and inflammatory response with complex pathogenesis. Over activation of the pyrin domain-containing protein 3 (NLRP3) inflammasome triggers the secretion of interleukin (IL)-1β and IL-18, induces pyroptosis, and promotes the release of a large number of pro-inflammatory proteins. All of which contribute to the development of NAFLD. There is a great deal of evidence indicating that plant-derived active ingredients are effective and safe for NAFLD management. This review aims to summarize the research progress of 31 active plant-derived components (terpenoids, flavonoids, alkaloids, and phenols) that alleviate lipid deposition, inflammation, and pyroptosis by acting on the NLRP3 inflammasome studied in both *in vitro* and *in vivo* NAFLD models. These studies confirmed that the NLRP3 inflammasome and its related genes play a key role in NAFLD amelioration, providing a starting point for further study on the correlation of plant-derived compounds treatment with the NLRP3 inflammasome and NAFLD.

## 1 Introduction

Non-alcoholic fatty liver disease (NAFLD), defined as the presence of substantial lipid deposits in the liver not resulting from alcohol consumption, covers a spectrum of disorders. These include non-alcoholic fatty liver (NAFL), non-alcoholic steatohepatitis (NASH), NASH-associated fibrosis, cirrhosis, and liver cancer. NAFLD has replaced viral hepatitis as the world’s most common liver disorder, accounting for about 25% of the global incidence of liver disease (Younossi et al., 2016). Patients with NAFLD have a significantly increased risk of cancer (Kanwal et al., 2018; Desai et al., 2019). Insulin resistance, nutritional imbalances, genetic factors, and intestinal microbiota induce the occurrence and development of NAFLD (Buzzetti et al., 2016). Increasing evidence indicates that inflammatory signaling pathways are associated in NAFLD progression. NLRP3 inflammasome acts as a sensor for multiple danger signals, which include damage-related molecular patterns (DAMPs) of endogenous or exogenous pathogen signals and pathogen-associated molecular patterns (PAMPs)-induced aseptic inflammation (Prochnicki et al., 2016). The NLRP3 inflammasome plays a key role in NAFLD development (Wu et al., 2015; Yang G. et al., 2016; Wei et al., 2019).

The pathogenesis of NAFLD is complex, and the pathogenesis and disease processes in different patients are diverse. It is known that the NLRP3 inflammasome plays a major role in inflammation because the inflammatory process is blunted under experimental conditions that inhibit the NLRP3 inflammasome (Di Virgilio 2007). It is expressed in hepatic hepatocytes, myofibroblasts, endothelial cells, epithelial cells, stellate cells, and Kupffer cells (KCs) (Negash and Gale 2015). Meanwhile, NLRP3 inflammasome-related components increase during liver damage (Ganz et al., 2011). At present, no drug has been approved by the Food and Drug Administration (FDA) for the treatment of NAFLD. A number of studies have shown that NLRP3 inflammasome-related inhibitors have therapeutic effects on NAFLD. For example, MCC950, the most widely studied inhibitor, specifically inhibits the assembly of the NLRP3 inflammasome and has a strong inhibitory effect on the inflammatory progression of NAFLD (Mridha et al., 2017). Many plant-derived active ingredients also inhibit NLRP3 inflammasome activation or assembly.

Plant-derived compounds exhibit a rich breadth of structures, diverse biological activities, and high safety. Many have lipid-lowering, hypoglycemic, anti-inflammatory, and hepatoprotective effects and show significant inhibitory activities on NLRP3 inflammasome, making them valuable resources for the development of new NAFLD drugs. Therefore, screening of NLRP3 inhibitors from plant-derived compounds to treat NAFLD has sparked wide interest in the scientific community. In this review, we discussed the plant-derived active ingredients that regulate the NLRP3 inflammasome to improve NAFLD *in vivo* and *in vitro*, including terpenoids, flavonoids, phenols, and alkaloids. This information may provide a reference for further research and innovative drug development for the prevention and treatment of NAFLD.

## 2 An overview of NLRP3 inflammasome

The NLRP3 inflammasome is one of the most significant multimeric protein complexes involved in immune system functions and is widely regarded as the key mechanistic effector of intracellular inflammation (Chen et al., 2015). It consists of the sensor moiety NLRP3, the adaptor protein ASC, and the effector protease caspase-1 (procaspase-1). NLRP3 is a multi-protein complex consisting of three domains: the central nucleotide-binding domain (NACHT), the carboxy-terminal leucine-rich repeat sequence (LRR), and the amino-terminal pyrin domain (PYD). NLRP3 contains a PYD, ASC contains PYD and CARD domains, which interact with ASC through PYD after activation, and the CARD domain of ASC recruits the CARD domain of procaspase-1, which contains CARD and caspase-1 domains in the inactive state. The tight connection of these three proteins forms the NLRP3-ASC-procaspase-1 complex, also known collectively as the NLRP3 inflammasome (Schroder et al., 2010). The precursor molecule procaspase-1 cleaved itself to form active caspase-1, including two subunits, p20 and p10, which promoted the shearing of pro-IL-1β and pro-IL-18 into mature IL-1β and IL-18, leading to inflammation (Haneklaus and O'Neill 2015). Although a moderate inflammatory response helps to resist the invasion of pathogenic microorganisms, the NLRP3 inflammasome can cause great damage to the body if it is over-activated, resulting in excessive release of cytokines such as IL-1β and IL-18, and the resulting “cytokine storm” inflammatory cascade.

Activation of the NLRP3 inflammasome involves initiation and activation steps. The initiation step (Signal 1) is provided by an inflammatory stimulant that stimulates the toll-like receptor (TLR) and then induces nuclear factor kappa-B (NF-κB) activation. NF-κB further promotes the expression of NLRP3, pro-IL-1β, and pro-IL-18. The activation step (Signal 2) includes ion channel opening caused by activation of purinergic receptor X7 (P2X7) on the cell surface or release of intracellular ATP, triggering intracellular K^+^ outflow and Ca^2+^ influx (Munoz-Planillo et al., 2013; Kelley et al., 2019), damaged mitochondria releasing large amounts of reactive oxygen species (ROS) (Cruz et al., 2007; Zhou et al., 2010; Zhou et al., 2011), phagocytic irritants such as amyloid-beta leading to lysosome rupture and cathepsin B release (Campden and Zhang 2019; Chevriaux et al., 2020; Svadlakova et al., 2020), which induce activation of the NLRP3 inflammasome. Activation of the NLRP3 inflammasome can lead to recruitment of ASC and caspase-1, promotion of self-oligomerization, self-shearing and activation of caspase-1, and lysis of pro-IL-1β and pro-IL-18 to form mature IL-1β and IL-18 and exert inflammatory effects (Shao et al., 2019).

## 3 The NLRP3 inflammasome and NAFLD

### 3.1 The NLRP3 inflammasome has been confirmed to be involved in the development of NAFLD

In recent years, the role of the NLRP3 inflammasome activation in NAFLD/NASH has received considerable attention. In a liver sample from 77 full-spectrum NAFLD patients, mRNA and protein levels of NLRP3 and IL-18 were significantly higher than those in non-NASH patients, and IL-1β mRNA expression was associated with collagen-1A1, a pro-fibrosis gene expressed by activated hepatic stellate cells (HSCs) (Wree et al., 2014). In another study, NLRP3 inflammasome-associated genes (ASC, caspase-1, and NLRP3) were significantly upregulated in patients with NASH (Csak et al., 2011).

In animal studies, hepatic NLRP3, ASC, procaspase-1, and pro-IL-1β mRNA expression increased in methionine-choline deficient diet (MCD)-fed mice (Csak et al., 2011). Interestingly, MCD-induced NASH in NLRP3−/− mice showed that serum alanine transaminase (ALT), aspartate transaminase (AST), IL-1β, and IL-18 levels were lower than those in non-knockout mice and significantly reduced hepatocyte inflammation, hepatomegaly, and liver fibrosis (Cai et al., 2017). Another study using NLRP3−/−, ASC−/−, and caspase-1−/− mice also found that hepatic steatosis and inflammation in NASH were indeed associated with activation of the NLRP3 inflammasome (Henao-Mejia et al., 2012). The NLRP3 inhibitor MCC950 can significantly block NLRP3 expression and ameliorate NASH pathology and lipid deposition in MCD-fed and foz/foz-induced NASH mouse models and hepatic NLRP3, procaspase-1, active caspase-1, pro-IL-1β, and IL-1β levels were significantly decreased (Mridha et al., 2017).

In *in vitro* experiments, MCC950 reduced IL-1β release by KCs and bone marrow-derived macrophages (BMDMs) and eliminated associated neutrophil migration (Mridha et al., 2017). NLRP3 deletion inhibited the upregulation of NLRP3, ASC, and caspase-1 mRNA and protein expression levels in palmitic acid (PA)-treated KCs (Csak et al., 2011). Similarly, in HepG2 and L02 cells, inhibition of NLRP3 activity reduced uric acid-induced lipid accumulation (Wan et al., 2016).

These clinical and basic studies suggest that activation of the NLRP3 inflammasome may promote the pathological progression of NAFLD.

### 3.2 NLRP3 inflammasome regulates NAFLD by mediating inflammatory response and pyroptosis

Activated NLRP3 inflammasome releases inflammatory particles and induces pyroptosis, which is significantly associated with NAFLD.

Studies have shown that activated NLRP3 inflammasomes result in caspase-1 activation (Qiu et al., 2018; Yu et al., 2019; Shi et al., 2020). Activated caspase-1 performs two important functions. First, it promotes the conversion of pro-IL-1β and pro-IL-18 to IL-1β and IL-18. The persistence of this process leads to the secretion of large amounts of IL-1β and IL-18 and the initiation of pyroptosis, all of which are major contributors to the progress of inflammatory diseases. These processes spread the inflammatory process to the outside of the cell and release a large number of inflammasome particles, which aggravate the inflammatory response of the liver and promote the occurrence of NAFLD (Henao-Mejia et al., 2012). Second, pyroptosis depends on the inflammasome-mediated activation of caspase-1. Membrane insertion of the pyroptosis medium Gasdermin D (GSDMD) leads to the formation of plasma membrane pores, causing intracellular protein release, ion decompensation, water influx, and cell swelling. Active caspase-1 promotes the cleavage of GSDMD, then GSDMD binds to phosphatidylserine and phosphatidylinositol on the cell membrane and forms membrane pores that signal the beginning of cell death. These pores allow the introduction of a large number of active inflammatory cytokines and DAMPs into the extracellular environment, where they are recognized by inflammatory cells and lead to pyroptosis (Swanson et al., 2019). That study found that the expression of GSDMD and its pyrogenic-induced fragment GSDMD-N were upregulated in NAFLD/NASH liver tissues, and the increased level of GSDMD-N protein in the liver was correlated with NAFLD activity score and fibrosis (Xu B. et al., 2018). This suggests a correlation between the expression of the NLRP3 inflammasome-GSDMD-pyroptosis triplet and the development of NASH.

**FIGURE 1:**
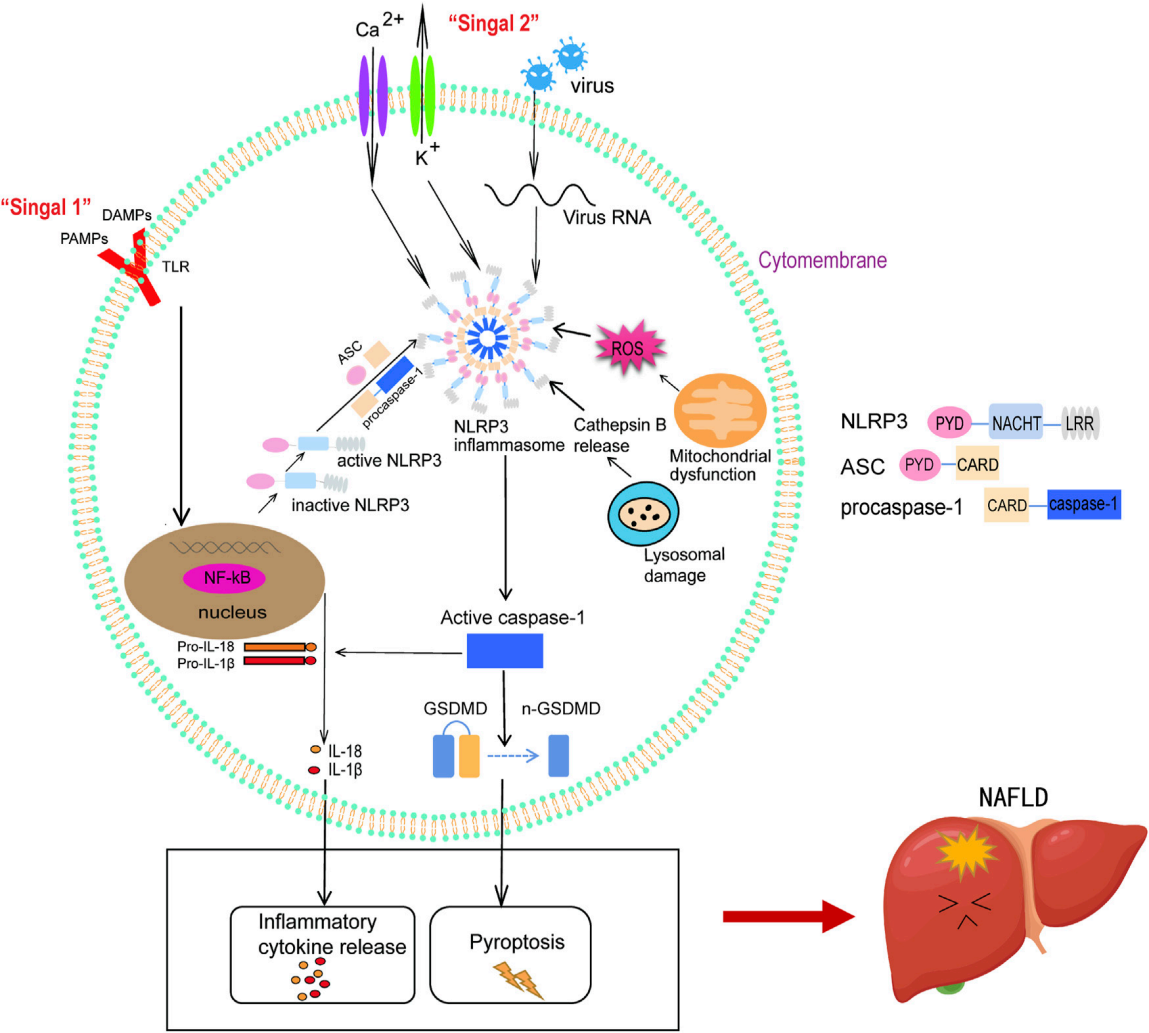
Activation of NLRP3 inflammasome mediates the development of NAFLD. The activation of NLRP3 inflammasome includes initiation and activation steps. During the initiation step, TLR is stimulated by extracellular signalling molecules, leading to NF-κB activation, which activates NLRP3 and promotes the expression of pro-IL-1β and pro-IL-18 (Signal 1). During the activation step, stimulation such as K^+^ outflow accompanied Ca^2+^ influx, ROS release, mitochondrial dysfunction, and lysosome damage induce the assembly of NLRP3 inflammasome (Signal 2). After NLRP3 inflammasome activation, activated caspase-1 converts pro-IL-1β and pro-IL-18 into mature IL-1β and IL-18, leading to the release of inflammatory cytokines. Activated caspase-1 dissociates GSDMD, releases its N-terminal, and forms pores in the plasma membrane, stimulating the occurrence of pyroptosis. The interaction between inflammatory factors and pyroptosis induces the inflammatory fibrosis microenvironment in the liver and promotes the occurrence and development of NAFLD.

Hepatocyte pyroptosis and the continuous release of inflammatory factors induce the formation of an inflammatory fibrotic microenvironment in the liver, which promotes the development of NAFLD (Gaul et al., 2021) (Figure 1).

## 4 Plant-derived active ingredients treat NAFLD by regulating NLRP3 inflammasome

Here, we described plant-derived active ingredients that improve NAFLD by inhibiting the NLRP3 inflammasome in a variety of cell and animal models and the mechanisms underlying these inhibitory effects. These active ingredients are mainly isolated or derived from plants or traditional Chinese herbs and can be roughly classified as terpenoids, flavonoids, alkaloids, and phenols (Table 1).

**TABLE 1:** Plant-derived bioactive compounds treat NAFLD by regulating NLRP3 inflammasome.

Classification	Active ingredients	Chemical formula	Experimental models	Effects on NAFLD	Regulation of NLRP3, inflammation, and pyroptosis	Mechanism for regulating NLRP3	References
Terpenoids	Antcin A	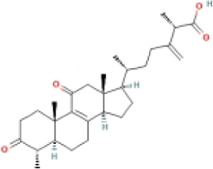	HFD-fed C57BL/6J mice	1. ALT, AST↓	1.NLRP3, ASC, and caspase-1↓	Inhibit the assembly and activation of NLRP3 inflammasome	Ruan et al. (2021)
				2. Hepatic lipid droplets↓	2.Serum IL-1β, IL-18, and TNF-α↓		
					3.GSDMD-FL, GSDMD-NT↓		
			KCs cells + LPS/Nigericin	—	1.NLRP3, caspase-1↓		
					2.IL-1β, IL-18, and TNF-α↓		
					3.GSDMD-NT↓		
	Ginseng Saponin Rh1/Rg2	Rh1 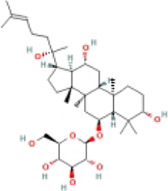 Rg2 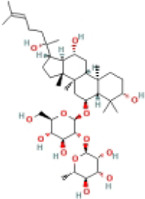	FFD-fed male C57BL/6 mice	1.Liver weight, liver size↓	1.NLRP3 inflammasome↓	Inhibit NLRP3 inflammasome activation by promoting mitophagy	Wang et al. (2021)
				2.ALT↓	2.IL-1β, ARG1, CCL2, CCL4, CXCL2, and TNF-α↓		
				3.Serum TC↓			
				4.Hepatic lipid droplets↓			
				5.Collagen deposition↓			
			ImKCs + LPS	—	1.NLRP3 inflammasome↓		
					2.IL-1β, ARG1, CCL2, and IL-10↓		
			Primary hepatocytes were isolated from the C57BL/6 mice + PA	1.Intracellular lipid droplets↓	—		
	Ginsenoside Rg1	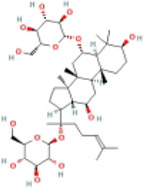	HFD-fed female C57BL/6 mice	1.Body weight, liver weight↓	1.NLRP3↓	Inhibit NLRP3 inflammasome activation	Wong et al. (2015)
				2.ALT, AST↓	2.Cleaved IL-1β, IL-18, and IL-1β↓		
				3.Serum TG↓			
				4.Adipose infiltration↓			
				5.Hepatic FFA↓			
	Andrographolide	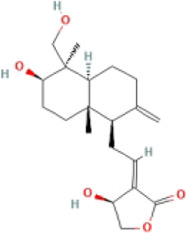	CDAA-fed male C57BL/6 mice	1.Body weight, liver weight↓	1.NLRP3, ASC, and caspase-1↓	Inhibit NF-κB/NLRP3 pathway	Cabrera et al. (2017)
				2.ALT↓	2.IL-1β↓, F4/80, MCP-1, TNF-α, IFNγ, and iNOS↓		
				3.Hepatic TC↓			
				4.NAS score↓			
				5.Heaptic Col1A1, α-SMA↓			
			HepG2 cells + PA + LPS	—	1.IL-1β, IL-6↓		
			Hepatocytes were obtained from pathogen-free male Wistar + LPS	—	1.Caspase-1↓		
					2.iNOS↓		
	14-Deoxy-11,12-Didehydroandrographolide	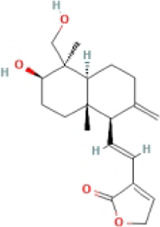	HFHC-fed male C57BL/6J mice	1.ALT, AST↓	1.NLRP3, caspase-1 ↓	Attenuate NLRP3 inflammasome activation	Liu et al. (2020a)
				2.Plasma TC↓	2.IL-1β, TNF-α↓		
				3.Hepatic lipid droplets↓			
	β-patchoulene	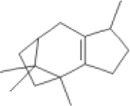	HFD-fed male SD rats	1.Body weight, liver index↓	1.NLRP3, ASC, and Cleaved-caspase-1↓	Suppress the TXNIP/NLRP3 inflammasome pathway	(Luo et al. (2021))
				2.ALT, AST↓	2.IL-18, IL-1β↓		
				3.Hepatic and Serum TC, TG↓			
				4.NAS scores↓, hepatic lipid droplets↓			
	Sweroside	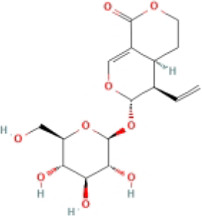	MCD-fed male C57BL/6 mice	1.ALT, AST↓	1.Caspase-1↓	Suppress NLRP3 inflammasome activation	Yang et al. (2020)
				2.Hepatic TG↓	2.IL-1β↓		
					3.Hepatic lipid droplets↓		
			BMDMs + LPS + ATP + nigericin	—	1.NLRP3, ASC, procaspase-1, and caspase-1↓2.Pro-IL-1β, IL-1β↓		

	Carnosic acid	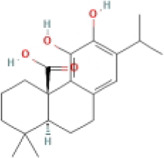	HFD-fed male C57BL/6 mice	1.ALT, AST↓	1.NLRP3, caspase-1↓	Suppress MARCKS/NLRP3 inflammasome signaling pathway	Song et al. (2018)
				2.Hepatic and serum TG, TC↓, FBG, plasma insulin↓	2.IL-1β, IL-18, TNF-α, IL-2, IL-4, IL-6, IL-12, and IFN-γ↓		
				3.Inflammatory score↓, Lipid droplets↓			
	Gardenoside	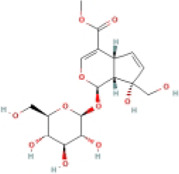	HFD-fed male C57BL/6J mice	1.ALT, AST↓	1.NLPR3, ASC, and caspase-1 p20↓	Inhibit the activation of NLRP3 inflammasome via the CTCF/DPP4 axis	Shen et al. (2021)
				2.Serum TG, TC↓	2.IL-1β, IL-18↓		
				3.Histological score↓	3.GSDMD-N↓		
			AML12 cells + PA + LPS	1.Intracellular TG↓	1.NLPR3, ASC, and caspase-1 p20↓		
				2.Intracellular Collagen-IV, Collagen-V↓	2.IL-1β, IL-18↓		
					3.GSDMD-N↓		
	Glycyrrhizin	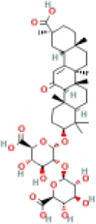	MCD-fed male C57BL/6 mice	1.Hepatic TG, TC, HDL-C↑	1.NLRP3, caspase-1, ASC, procaspase-1, and cleaved caspase-1↓	Suppress the TLR/NLRP3 inflammasome activation	Yan et al. (2018)
				2.Hepatic lipid droplets↓, NAFLD activity, steatosis scores and inflammation score↓	2.Pro–IL-1β, cleaved IL-1β, IL-1β, and TNF-α↓		
				3.α-SMA, ColA1, ColA2↓			
Flavonoid	Naringenin	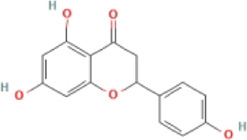	MCD-fed male C57BL/6 mice	1.ALT, AST↓	1.NLRP3↓	Downregulate the NF-κB/NLRP3 pathway	Wang et al. (2020a)
				2.Hepatic TG↓	2.IL-1β, IL-18↓, TNF-α, CD68, and CD64↓		
				3.Lipid droplets↓			
			HepG2 cells + LPS + OA	1.Intracellular TG↓	1.NLRP3↓		
				2.Lipid droplets↓	2.IL-1β, IL-18↓		
			Primary hepatocytes were isolated from male C57BL/6 or NLRP3^−/−^ mice + LPS + OA	1.Lipid deposition in WT hepatocytes↓, was not effective in NLRP3^−/−^ hepatocytes	1.NLRP3, IL-1β expression in WT hepatocytes↓, not effective in NLRP3^−/−^ hepatocytes		
			Primary liver KCs were isolated from C57BL/6 mice + LPS	—	1.NLRP3↓		
	Apigenin	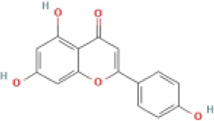	HFD-fed male C57BL/6 J mice	1.Body weight, liver weight↓	1.NLRP3, ASC, procaspase-1, and caspase-1↓	Inhibit the XO/NLRP3 pathways	Lv et al. (2019)
				2.ALT, AST↓	2.Hepatic and serum IL-1β, IL-18↓		
				3.Serum TG, TC↓, insulin sensitivity↑, blood glucose↓			
			Hepa1–6 cells + FFA	1.Intracellular TG↓	1.NLRP3, ASC, and caspase-1↓		
				2. Lipid droplets↓			
	Echinatin	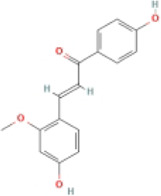	MCD-fed C57BL/6 mice	1.Body weight↓	1.Caspase-1↓	Disrupt the association of HSP90 and NLRP3 complex	(Xu et al. (2021))
				2.ALT, AST↓	2.IL-1β, TNF-α↓		
				3.Hepatic lipid droplets↓			
				4.α-SMA, Col1A1↓			
			LPS-primed BMDMs	—	1.NLRP3, caspase-1, and ASC↓		
					2.Pro-IL-1β, IL-1β↓		
	Licochalcone E	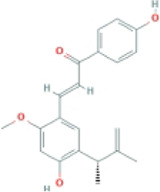	HepG2 cells + PA	1.Intracellular TG, TC↓, glucose consumption↑	1.NLRP3, caspase-1↓	Inhibit the NLPR3 signaling pathway	Cao et al. (2021)
					2.Pro-IL-1β, IL-1β, IL-18, and TNF-α↓		
	Silybin	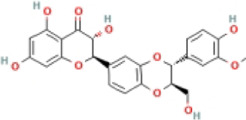	HFD-fed male C57BL/6 mice	—	1.NLRP3, caspase-1↓	Inhibit the NAD+/SIRT2/NLRP3 inflammasome pathway	Zhang et al. (2018)
					2.IL-1β↓		
			HepG2 cells + PA	—	1.NLRP3, ASC↓		
					2.IL-1β↓		
			Primary mouse hepatocytes + PA	—	1.NLRP3, ASC↓		
					2.IL-1β↓		
	Baicalin	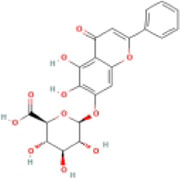	AML-12 cells + PA	—	1.NLRP3↓	Inhibit TXNIP/NLRP3 signaling axis	Zhang et al. (2017a)
					2.IL-1β, IL-6↓		
	Cyanidin-3-O-glucoside	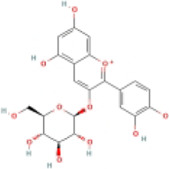	Primary human hepatocytes from NAFLD patients	1.Intracellular TG, blood glucose, and HOMA-IR↓	—	Inhibit NLRP3 inflammasome activation	Zhu et al. (2012)
			HFD-fed male mice	1.Body weight, liver weight, and liver index↓	1.NLRP3, procaspase-1, and caspase-1↓		
				2.Hepatic TG↓	2.IL-1β, IL-18↓		
				3.Hepatic lipid droplets↓			
	Mangiferin	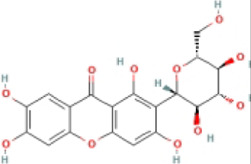	HFD-fed male C57BL/6J mice	1.Body weight, liver weight↓	1.NLRP3, procaspase-1, and caspase-1 (p10) ↓	Inhibit NLRP3 inflammasome activation	Yong, Ruiqi et al. (2021)
				2.ALT, AST↓	2.IL-1β↓		
				3.Hepatic TG↓, serum FBG, TG, LDL-c, TC↓, and HLD-c↑, insulin resistance, glucose tolerance ↑	3.GSDMD-N↓		
				4.Steatosis scores↓			
			HepG2 cells + PA	1.Intracellular TG↓, glucose consumptions↑	1.NLRP3, procaspase-1, and caspase-1 (p10) ↓		
					2.IL-1β↓		
					3.GSDMD-N↓		
	Nobiletin	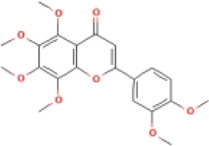	AML-12 cells + PA	—	1.NLRP3, caspase-1↓	Suppress NLRP3 inflammasome activation in a SIRT1-dependent manner	Peng, Li et al. (2018)
					2.IL-1β, IL-18↓		
Alkaloids	Berberine	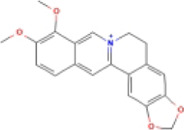	HFD-fed male SD rats	1.Body weight↓	1.NLRP3, caspase-1↓	Inhibit NOD/NLRP3 inflammasome axis	Mai et al. (2020), Wang et a al., (2020b)
				2.ALT, AST↓	2.IL-1β, IL-18, TNF-α, and IL-6↓		
				3.Serum TG, TC↓			
				4.Hepatic fat vacuoles↓			
			MCD-fed male C57BLKS/J mice	1.ALT, AST↓	1.NLRP3, caspase-1↓	Suppress NLRP3 inflammasome activation and pyroptosis via the ROS/TXNIP *Axis*	
				2.Hepatic lipid droplets↓	2.TNF-α↓		
			AML12 cells + PA + LPS	1.Intracellular lipid droplets↓	1.NLRP3, procaspase-1, and caspase-1↓		
					2.GSDMD-N↓		
	Demethylenetetrahydroberberine	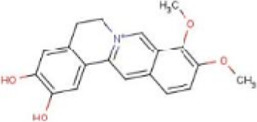	MCD-fed male C57BL/6 mice	1.Liver index↓	1.NLRP3, ASC, cleaved caspase-1, and procaspase-1↓	Target NLRP3 and inhibit the NLRP3 inflammasome activation	Zhang et al. (2021)
				2.ALT, AST↓	2.IL-1β, TNF-α, and IL-6↓		
				3.Hepatic TG, TC↓			
				4.Hepatic fat vacuole↓			
				3.Hepatic α-SMA, collagen -1A1↓			
	Matrine	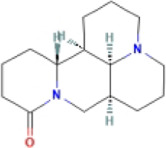	MCD-fed male C57BL/6J mice	1.ALT, AST↓	1.NLRP3↓	Downregulate the expression of NLRP3	Mahzari et al. (2019)
				2.Hepatic TC↓	2.TNF-α↓		
				3.Hepatic collagen-1↓			
	Betaine	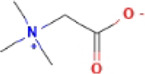	Fructose-fed male SD rats	1.Body weight↓	1.NLRP3, ASC, and caspase-1↓	Suppress the NF-κB/NLRP3 inflammasome pathway	Ge et al. (2016)
				2.Hepatic and Serum TG, TC ↓, glucose levels↓	2.IL-6, IL-1β↓		
Phenols	Cannabidiol	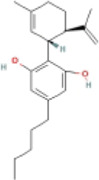	HFC-fed male C57BL/6J mice	1.ALT, AST↓	1.NLRP3, caspase-1↓	Regulate the NF-κB/NLRP3 inflammasome pathway	Huang et al. (2019); Jiang et al. (2021)
				2.Hepatic and Serum TG, TC↓	2.IL-1β, TNF-α↓		
				3.Inflammation scores↓			
			RAW264.7 + ATP + LPS	—	1.NLRP3, caspase-1↓		
					2.IL-1β, TNF-α, and IL-6↓		
	Salvianolic acid A	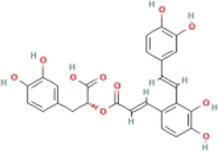	HFD-fed male SD rats	1.Body weight, liver index↓	1.NLRP3, ASC, and caspase-1↓	Inhibit the TXNIP/NLRP3 pathway	Ding et al. (2016)
				2.Serum ALT, AST↓	2.IL-1β, TNF-α, and IL-6↓		
				3.Hepatic and serum TG, TC↓			
				4.Hepatic lipid droplets, inflammation score↓			
			HepG2 cells + PA	1.Intracellular lipid droplets↓	1.NLRP3, ASC, and caspase-1↓		
					2.IL-1β, TNF-α, and IL-6↓		
	Dieckol	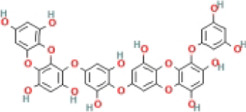	HFD-fed male C57BL/6N mice	1.Hepatic TG↓	1.NLRP3, ASC, caspase-1, and cleaved caspase-1↓	Inhibit the NLRP3 inflammasome/pyroptosis	Oh et al. (2021)
				2.Hepatic macrovesicular steatosis, lobular inflammation, and ballooning↓	2.GSDMD, cleaved-GSDMD↓		
				3.Hepatic FFA↓			
	Polydatin	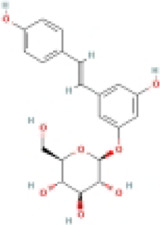	Fructose-fed male SD rats	1.Hepatic TG, TC↓	1.NLRP3, ASC, and caspase-1↓	Suppress the TXNIP/NLRP3 inflammasome pathway	Zhao et al. (2018)
			Buffalo rat liver cells + fructose	1.Intracellular TG, TC↓	2.IL-1β, TNF-α↓		
			HepG2 cells + fructose	1.Intracellular TG, TC↓			
	Resveratrol	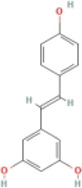	HFD-fed male C57BL/6 J mice	1.Liver weight↓	1.NLRP3, ASC, and caspase-1↓	Suppress the SIRT/NLRP3 inflammasome pathway	Yang and Lim (2014)
				2.Hepatic and serum TG↓, serum adiponectin, glucose, and HOMA-IR↓	2.IL-1, IL-6, and TNF-α↓		
	Magnolol	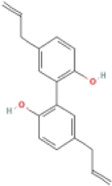	Tyloxapol-injected male Wistar rats	1.Plasma TG, TC↓	1.NLRP3, ASC, and caspase-1↓	Inhibit the NLRP3 inflammasome activation	Kuo et al. (2020)
			HepG2 cells + PA	1.Intracellular TG↓	2.IL-1β, IL-18, IL-6, and TNF-α↓		
				2.Lipid droplets↓			
	Salidroside	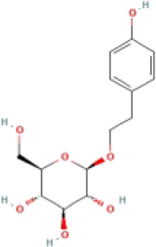	HFD-fed male C57BL/6 mice	1.Body weight, liver weight↓	1.NLRP3, procaspase-1, and caspase-1↓	Regulate the TXNIP/NLRP3 signaling pathway	Zheng et al. (2018)
				2.Serum TG, TC, LDL-C↓, HDL-C↑, FBG, and HOMA-IR↓	2.Pro-IL-1β, IL-1β, IL-18, and TNF-α↓		
				3.Hepatic steatosis, inflammation, ballooning, and NAFLD activity scores↓			
			Primary hepatocytes were isolated from C57BL/6 mice + high glucose plus insulin	1.Intracellular lipid content↓			
				2.Insulin sensitivity↑			

### 4.1 Terpenoids

Terpenoids are natural products with a wide distribution, diverse structures, and various biological activities. They are composed of isoprene and play important roles in the metabolism of all organisms (Bouvier et al., 2005). Terpenoids have been proven to have antioxidant and anti-inflammatory effects and can be used as therapeutic drugs for many inflammatory diseases (Grassmann 2005; Kim et al., 2020). Many plant-derived terpenoids exert anti-NASH effects by inhibiting NLRP3 inflammasome activation.

#### 4.1.1 Antcin A

Antcin A, a terpenoid isolated from *Antrodia camphorata*, is a classical, traditional Chinese medicine used for NAFLD therapy. Antcin A has strong anti-inflammatory and hepatoprotective effects (Xu G. B. et al., 2018). *In vivo* and *in vitro* studies have shown that antcin A could ameliorate liver lipid deposition, reduce ALT and AST levels, and inhibit the expression levels of NLRP3 inflammasome (including NLRP3, ASC, and caspase-1) and pyroptosis-associated proteins (GSDMD-FL and GSDMD-NT). The cell membrane pore permeability and the number of pyroptotic cells significantly decreased after treatment with Antcin A. It worked by binding to NLRP3 and inhibiting the assembly and activation of the NLRP3 inflammasome, further mediating the inflammatory response and pyroptosis and thereby improving NASH (Ruan et al., 2021).

#### 4.1.2 Ginseng saponins, Rh1/Rg2/Rg1

Ginseng saponins can be divided into Rg1, Re, Rg2, Rh1, Rf, Rb1, Rb2, Rd, Rg3, Rh2, and Ro. Substantial evidence suggests that ginseng saponins have antioxidant, anti-inflammatory, and anti-pyroptosis properties (Wong et al., 2015; Xu Y. et al., 2018). Rh1/Rg2 extenuated hepatic lipid and fibrosis and reduced lipogenesis-related, fibrosis-related, and inflammatory genes in fast food diet (FFD)-fed male C57BL/6J mice. In addition, Rh1/Rg2 inhibited the NLRP3 inflammasome and the release of IL-1β. *In vitro* experiments have shown the exertion of anti-NASH effects by maintaining mitochondrial homeostasis and inhibiting NLRP3 inflammasome activation (Wang et al., 2021). Another study showed that ginsenoside Rg1 also alleviated this phenotype and reduced NLRP3, IL-1β, and cleaved IL-1β expression in NASH model mice by inhibiting inflammasome activation (Xu B. et al., 2018).

#### 4.1.3 Andrographolide (Andro)

Andro, a natural terpenoid from *Andrographis paniculata*, possesses a wide range of bioactive compounds, including anti-inflammatory, antitumor, anti-diabetic, anti-bacterial, anti-malarial, and hepatoprotective properties (Lim et al., 2012; Jayakumar et al., 2013). *In vivo* experiments have shown that it could reduce liver fat content, alleviate the expression of liver fibrosis indicators and autophagy, and inhibit the expression of NLRP3, ASC, caspase-1, and IL-1β. *In vitro* studies also have detailed good anti-inflammatory and anti-fibrosis effects of Andro and its anti-NASH effects were mainly regulated by NF-κB-dependent NLRP3 inflammasome (Cabrera et al., 2017). One study found that the oral bioavailability of 14-Deoxy-11, 12-Didehydroandrographolid, a derivative of Andro was higher than Andro (Guan et al., 2011; Chen et al., 2014). It could lower ALT and hepatic total cholesterol (TC) levels and reduce the expression of NLRP3 and related inflammatory factors by increasing antioxidant and anti-inflammatory activities in NAFLD mice (Liu Y. T. et al., 2020).

#### 4.1.4 β-patchoulene (β-PAE)

β-PAE, a natural terpenoid of *Pogostemonis herba*, has anti-inflammatory, antioxidant, and hepatoprotective effects (Wu et al., 2018; Liu Y. et al., 2020). Studies have shown that β-PAE ameliorates serum lipid and liver transaminase in a high-fat diet (HFD)-induced NASH model. In addition, β-PAE reduced the expression of thioredoxin interaction protein (TXNIP), NLRP3, ASC, and cleaved-caspase-1 and inhibited the release of IL-Iβ. TXNIP is a key modulator of the redox system that inhibits TXN (thioredoxin) to cause cellular oxidative stress (Zhou et al., 2010). β-PAE reduced the levels of ROS and TXNIP, futher inhibited NLRP3 inflammasome activation (Luo et al., 2021).

#### 4.1.5 Sweroside

Sweroside, a plant-derived terpenoid, can effectively alleviate liver fibrosis and liver injury-related diseases (Yang Q. L. et al., 2016; Gong et al., 2020) and has been shown to delay the pathological progression of NASH, such as liver inflammatory, lipid accumulation, and fibrosis *in vivo*. This was accompanied by inhibitory effects on the hepatic NLRP3 inflammasome. Additionally, it has been shown to inhibit inflammasome activation as NLRP3, ASC, procaspase-1, caspase-1, and IL-1β reduced in the BMDMs inflammatory model induced by lipopolysaccharide (LPS), adenosine triphosphate (ATP), and nigericin (Yang et al., 2020). These results indicated that targeting the NLRP3 inflammasome with sweroside could be beneficial for improving NASH symptoms.

#### 4.1.6 Carnosic acid (CA)

CA is a natural terpenoid isolated from rosemary and common sages. It has been shown that CA can reduce TC, triglyceride (TG), ALT, AST, and insulin resistance, inhibit the expression of pro-inflammatory factors and adipogenic genes, and improve the expression of myristoylated alanine-rich protein kinase C substrate (MARCKS). MARCKS is a major protein kinase C (PKC) substrate in many different cell types, which has been investigated in LPS-induced disease from inflammation-related signaling pathways (Yin et al., 2016). The protein expression of NLRP3 and caspase-1 were upregulated in the MARCKS-deficient mice. MARCKS deficiency induced pro-inflammatory cytokine secretion, including IL-1β, IL-18, and tumor necrosis factor (TNF)-α, and induced NLRP3 inflammasome activation *in vivo*. This study suggested that the mechanism of CA treatment for NASH is to inhibit the activation of the NLRP3 inflammasome signaling pathway by increasing the expression of MARCKS (Song et al., 2018).

#### 4.1.7 Gardenoside

Gardenoside is the most effective bioactive molecule of the medicinal plant *Gardenia jasminoides Ellis* and has been shown to inhibit inflammatory cytokines and hepatocyte steatosis induced by free fatty acids (FFA), which play an important role in NAFLD (Liang et al., 2015). Gardenoside can ameliorate lipid deposition and liver fibrosis, and has been shown to decrease the expression levels of dipeptidyl peptidase-4 (DPP4), CCCTC-binding factor (CTCF), NLRP3, ASC, caspase-1 p20, GSDMD-N, and IL-1β *in vitro* and *in vivo* NAFLD models. DPP4, an aminopeptidase, regulates the activity of a variety of biological peptides and participates in a variety of pathological and physiological processes (Lambeir et al., 2003). CTCF is a gene known for its chromatin-organizing and transcription factor properties (Braccioli and de Wit 2019). Importantly, CTCF or DPP4 silencing showed effects similar to those of Gardenoside treatment, whereas CTCF overexpression offset this trend. Compared with the NAFLD model group, the number of apoptotic cells decreased and the cell membrane integrity of hepatocytes improved significantly in the gardenoside group. These results suggested that gardenoside inhibited the activation of NLRP3 inflammasome -mediated inflammation and pyroptosis *via* the CTCF/DPP4 axis (Shen et al., 2021).

#### 4.1.8 Glycyrrhizin (GL)

GL, derived from the traditional Chinese medicine licorice, is widely used for treating liver disease (Wang H. et al., 2017). In an MCD-induced NAFLD model study, GL reduced the lipid levels in the liver and serum, reduced the expression of apoptosis marker genes, regulated FFA synthesis, and inhibited liver fibrosis. The expression levels of TLR, NLRP3, ASC, caspase-1, and IL-1β were reduced after treatment with GL. This study confirmed that GL plays a therapeutic role in NASH by inhibiting the TLR/NLRP3 inflammasome signalling pathway (Yan et al., 2018).

### 4.2 Flavonoids

Flavonoids are naturally existing and broadly distributed secondary metabolites of plants with a variety of biological functions, including antibacterial, antioxidant, anti-inflammatory, and anti-fibrosis activities. Flavonoids can inhibit the production of inflammatory mediators such as IL-1β, IL-18, TNF-α, and IL-6 (Yi, 2018). An increasing number of flavonoids have been reported to show anti-inflammatory and anti-fibrotic activities through the inhibition of NLRP3 inflammasome activation.

#### 4.2.1 Naringenin

Naringenin is mainly found in plants, especially citrus fruits such as grapefruit (Orhan, Nabavi et al., 2015), and has anti-inflammatory (Jin et al., 2017), antioxidant (Zhao et al., 2017), lipid-lowering (Mulvihill et al., 2009), and immunomodulatory effects (Zeng et al., 2018). Naringenin reduced lipid accumulation and inflammation in the liver of MCD-induced mice and decreased the expression of NF-κB, NLRP3, IL-1β, and IL-18, but did not further extenuate lipid accumulation in the livers of NLRP3−/− mice. Meanwhile, similar results have been obtained *in vitro*. The study concluded that naringenin prevents NAFLD by downregulating the NF-κB/NLRP3 signaling pathway (Wang Y. et al., 2020).

#### 4.2.2 Apigenin (API)

API, a naturally existing bioflavonoid, is commonly found in fruits, herbs, and vegetables (Miean and Mohamed 2001). API has distinct pharmacological activities, such as antioxidant, anti-inflammatory activity (Lee et al., 2007), antitumor (Shao et al., 2013), and antidepressant effects (Li et al., 2016). Many studies have indicated that API has anti-obesity and anti-diabetic activity (Ono and Fujimori 2011; Kim et al., 2014). The potential mechanism of these therapeutic effects was attributed to the regulation of xanthine oxidase (XO) by API, which further regulated NLRP3 inflammasome activation and the release of inflammatory cytokines IL-1β and IL-18. These results indicated that API ameliorated HFD-induced NAFLD by regulating the XO/NLRP3 pathway (Lv et al., 2019).

#### 4.2.3 Echinatin (Echi)

Echi, a bioactive flavonoid of *Glycyrrhiza* plants (licorice), has antioxidant and anti-inflammatory activities (Fu et al., 2013; Tian et al., 2016). Echi ameliorated lipid deposition, fibrosis, and inflammation in an MCD-induced NASH mouse model. This study showed that Echi treatment had a beneficial effect on the level of ALT, AST, caspase-1, IL-1β, TNF-α, and lipid steatosis and fibrosis in the liver in MCD-induced NASH, which is similar to NLRP3 inhibitors, MCC950. These findings suggested that Echi could alleviate liver inflammation and the NASH pathology via inhibition of NLRP3 inflammasome. Further research showed that Echi exerts its inhibitory effect on the NLRP3 inflammasome by binding to heat-shock protein 90 (HSP90), a molecular chaperone family member that is crucial for the correct folding of many newly synthesized peptides and rematuration of denatured/misfolded client proteins, suppressing the ATPase activity of NLRP3 (Nizami et al., 2021), and destroying the association between cochaperone solanidine galactosyltransferases (SGT1) and HSP90-NLRP3 (Xu et al., 2021).

#### 4.2.4 Licochalcone E

Licochalcone E, a flavonoid isolated from licorice, possesses various biological activities, especially anti-diabetic effects (Park et al., 2012). Licochalcone E significantly raised the consumption of glucose and reduced the concentrations of TC and TG in PA-treated HepG2 cells. Further, licochalcone E depressed the expression of NLRP3, caspase-1, and IL-1β. Meanwhile, molecular simulations exhibited that licochalcone E has a high binding affinity for the NLPR3 inflammasome and inhibits the NLPR3 signalling pathway (Cao et al., 2021).

#### 4.2.5 Silybin

Silybin, a natural flavonoid from milk thistle (silybum marianum), has been shown to have significant anticancer, anti-diabetic, and beneficial liver effects (Ferenci et al., 1989; Huseini et al., 2006; Hawke et al., 2010; Serviddio et al., 2014). The present study demonstrated that silybin reduced NLRP3, ASC, and the secretion of IL-1β. The sirtuin type2 (SIRT2) protein is the third type of nicotbambeadenbe dinuclotide (NAD+) dependent histone deacetylases, and its activity and expression level are closely related to intracellular NAD + content (Wang et al., 2019). The NAD+/SIRT2 pathway is a crucial mediator of silybin blocking NLRP3 inflammasome activation in NAFLD, and the anti-inflammatory effects of silybin were impeded by silencing SIRT2 or the SIRT2 inhibitor AGK2. These results suggested that silybin constrains NLRP3 inflammasome assembly through the NAD+/SIRT2 pathway in mice with NAFLD (Zhang et al., 2018).

#### 4.2.6 Baicalin (BA)

BA, a primary bioactive flavonoid ingredient that originates from the root of *Scutellaria baicalensis Georgi*, has anti-inflammatory and antioxidant properties and extenuates cell lipotoxicity (Zhang Y. et al., 2017; Kwak et al., 2017). This study showed that BA inhibited the expression of NLRP3 and decreased the production of IL-1β and IL-6. A further mechanistic study indicated that BA alleviated PA-induced cytotoxicity in AML-12 cells via the inhibition of ER stress and TXNIP/NLRP3 inflammasome activation (Zhang J. et al., 2017).

#### 4.2.7 Cyanidin-3-O-glucoside (C3G)

C3G, one of the most plentiful anthocyanins belonging to the flavonoid family (Galvano et al., 2004), ameliorates oxidative stress and hepatic steatosis in mice (Guo et al., 2012). C3G lessened body weight, liver weight, TG, and lipid droplets in HFD-induced NAFLD. Meanwhile, C3G reduced the expression of NLRP3, caspase-1, IL-1β, and IL-18. Mechanistic studies showed that C3G promoted PINK1 (PTEN-induced kinase 1)/Parkin expression and increased PINK1-mediated mitophagy. The PINK1/Parkin pathway is considered to be the main pathway of mitochondrial autophagy (Nguyen et al., 2016). The knockdown of hepatic PINK1 canceled the mitophagy-inducing effect of C3G, which blunted the useful effects of C3G on oxidative stress, NLRP3 inflammasome activation, glucose metabolism, and hepatic steatosis (Zhu et al., 2012).

#### 4.2.8 Mangiferin (Man)

Man is particularly abundant in the leaves of mangoes and has anti-oxidative, anti-inflammatory, anticancer, and anti-diabetic activities (Yon, Teramoto et al., 2005; Gold-Smith, Fernandez et al., 2016; Saha, Sadhukhan et al., 2016). A study indicated that body weight, liver weight, TC, TG, ALT, and AST were reduced after treatment with Man, while NLRP3, caspase-1, IL-1β, and GSDMD expression were downregulated *in vivo* and *in vitro*. These results indicated that the anti-NAFLD activity of Man is mediated by its anti-inflammatory effects via NLRP3 inflammasome inhibition (Yong, Ruiqi et al., 2021).

#### 4.2.9 Nobiletin (Nob)

Nob, a polymethoxylated flavonoid present in citrus peels, has antioxidant, antitumor, and anti-lipotoxic properties (Nemoto, Ikeda et al., 2013; Gajaria, Patel et al., 2015; Kwon, Jung et al., 2015; Peng, Li et al., 2018). Nob decreased the expression of NLRP3, caspase-1, IL-1β, and IL-18 in AML-12 cells. Furthermore, Nob improved PA-induced lipotoxicity by inhibiting NLRP3 inflammasome activation in a SIRT1-dependent manner (Peng, Li et al., 2018).

### 4.3 Alkaloids

Alkaloids are widely distributed in various plants and are a kind of organic secondary metabolites containing nitrogen with various structures, which have a wide variety of bioactive roles, including antioxidant, anti-inflammatory, antitumor, and immunoregulatory activities (Liu, Yang et al., 2019). Several studies have shown that the effects of alkaloids on NASH were mediated by the inhibition of NLRP3 inflammasome activation.

#### 4.3.1 Berberine (BBR)

BBR is an isoquinoline alkaloid that is derived from diverse herbal plants, including *Coptis chinensis*. Studies have shown that BBR has anti-lipogenic, anti-inflammatory, anti-fibrotic, and antitumor effects (Luo, Tian et al., 2019). BBR significantly reduced ALT, AST, TC, and TG levels. BBR works by enhancing the permeability of intestinal mucosa and balancing intestinal innate immune constituents. Research has shown that the expressions of NOD1, NOD2, NLRP3, caspase-1, and the release of IL-1β, IL-18, and TNF-α were upregulated in NAFLD intestine and reduced after treatment of BBR. The mechanism may be inhibition of the NOD/NLRP3 inflammasome axis (Wang Q. et al., 2020). Another study suggested that BBR significantly ameliorated lipid accumulation, lipid peroxides, TNF-α expression, inhibited NLRP3 expression, caspase-1 activity, and GSDMD-N expression with a decrease in ROS levels and TXNIP expression *in vivo* and *in vitro*. These results suggested that BBR suppressed NLRP3 inflammasome activation and pyroptosis *via* the ROS/TXNIP axis to ameliorate NASH (Mai et al., 2020).

#### 4.3.2 Dimethylenetetrahydroberberine (DMTHB)

DMTHB, an innovative berberine derivative, has a similar structure to dimethyleneberberine (DMB), a natural mitochondria-targeting antioxidant that exhibits significant anti-inflammatory and antioxidant pharmacological activities (Zhang et al., 2015; Qiang et al., 2016). DMTHB suppressed AST and ALT, hepatic TG and TC, and indicators related to hepatic fibrosis in the MCD-fed model. In addition, further results have shown that DMTHB inhibited the release of IL-1β by suppressing the expression of NLRP3, ASC, and caspase-1. The anti-NASH mechanism was regarded to involve DMTHB, which could target the NLRP3 inflammasome to suppress NLRP3 inflammasome activation (Zhang et al., 2021).

#### 4.3.3 Matrine (Mtr)

Mtr is originally derived from the plant *Sophora flavescens* and possesses potent hepatoprotective (Liu et al., 2003) and anti-inflammatory effects (Zhang et al., 2011). It was verified that Mtr reduced AST, ALT, and collagen-1 and inhibited the expression of hepatic TNF-α and NLRP3 in the MCD-fed model. The anti-NASH was related to the downregulation of NLRP3 in the NASH model (Mahzari et al., 2019).

#### 4.3.4 Betaine

Betaine, a naturally occurring compound in common foods, has been used to treat NAFLD (Sookoian et al., 2017; Veskovic et al., 2019; Veskovic et al., 2020; Chen et al., 2021). Betaine could lower serum lipid, hepatic lipid, and blood glucose and reduce the levels of NLRP3, ASC, caspase-1, and IL-1β. Betaine ameliorated hepatic lipid accumulation, gluconeogenesis, and inflammation by inhibiting the activation of the hepatic NF-κB/NLRP3 inflammasome pathway in fructose-fed NAFLD rats (Ge et al., 2016).

### 4.4 Phenols

Phenolic compounds are secondary metabolites synthesized in the process of plant growth as important plant components. They have excellent lipid-lowering and anti-inflammatory properties, improve mitochondrial function, effectively inhibit lipid oxidation, and treat NASH and other inflammatory diseases (Lee et al., 2011; Rafiei et al., 2019).

#### 4.4.1 Cannabidiol (CBD)

CBD, a non-psychoactive compound extracted from *Cannabis sativa*, possesses a wide range of pharmacological functions, including antioxidant and anti-inflammatory functions, cardiac and neural protection, and anti-epileptic potential (Yang et al., 2014; Wang Y. et al., 2017). A previous study showed that CBD alleviated hepatic steatosis and oxidative stress and significantly decreased the expression of NF-κB, NLRP3, ASC, procaspase-1, caspase-1 p20, GSDMD, and cleaved GSDMD both *in vivo* and *in vitro*. The anti-NASH mechanism of CBD was related to the suppression of the NF-κB/NLRP3 inflammasome pathway (Huang et al., 2019; Jiang et al., 2021).

#### 4.4.2 Salvianolic acid A (SalA)

SalA, a highly efficacious phenolic acid, is a water-soluble compound isolated from *Radix Salvia miltiorrhiza*. Many studies have shown that SalA exerts various pharmacological properties, such as antioxidant, anti-inflammatory, anti-fibrotic, anti-carcinogenic (Zhang et al., 2014; Dai et al., 2015; Wang et al., 2015), and anti-apoptotic properties (Qiang et al., 2014). It reduced the levels of hepatic pro-inflammatory cytokines (IL-1β, TNF-α, and IL-6) and lipid accumulation (TG and TC) in the NAFLD model. This study concluded that TXNIP and NLRP3 inflammasome activation is involved in the pathogenesis of NAFLD. SalA extenuated hepatic lipid accumulation and inflammation in HFD-induced NAFLD, and these protective effects were associated with the TXNIP/NLRP3 pathway (Ding et al., 2016).

#### 4.4.3 Dieckol (DK)

DK, a natural phenolic compound found in *Laminaria japonica*, decreases hepatic steatosis by alleviating hepatic inflammation (Byun et al., 2021). The accumulation of TG and histological NAFLD score in the liver were reduced by DK in the HFD-induced NAFLD model. In addition, DK could decrease the expression of NLRP3, ASC, caspase-1, GSDMD, and cleaved-GSDMD to improve NAFLD *in vivo.* The pyroptotic cell numbers in the liver were increased by HFD and decreased after treatment with DK. Above all were related to decreased formation of the NLRP3 inflammasome and inhibition of pyroptosis (Oh et al., 2021).

#### 4.4.4 Polydatin

Polydatin is extracted from *Polygonum cuspidatum* (Siebold & Zucc) and has been used in traditional Chinese medicine to prevent liver disorders associated with oxidative stress, inflammation, and lipid deposition in patients and experimental animals (Li et al., 2018; Chen et al., 2019). Polydatin downregulated NLRP3, ASC, caspase-1, IL-1β, TG, and TC in the livers of fructose-fed rats. It was verified that the mechanism for NASH involves blocking ROS-driven TXNIP to suppress NLRP3 inflammasome activation (Zhao et al., 2018).

#### 4.4.5 Resveratrol

Resveratrol, a polyphenol present in grapes, mulberries, and red wine, exerts antioxidant and anti-inflammatory effects (Szkudelski and Szkudelska 2011). Resveratrol significantly reduced serum glucose and TG content. In addition, resveratrol treatment reduced the levels of pro-inflammatory markers such as IL-1, IL-6, and TNF-α. Futher mechnism research showed that these improvements were related to suppresse the SIRT/NLRP3 inflammasome pathway (Yang and Lim 2014).

#### 4.4.6 Magnolol (MG)

MG, a major bioactive compound isolated from *Magnolia officinalis*, has been shown to possess anti-inflammatory, anti-oxidative, and hepatoprotective properties (Tian et al., 2018; Zhang et al., 2019). MG reduced serum TG and TC content, decreased the expression of NLRP3, ASC, and caspase-1, and reduced the production of IL-1β, IL-18, IL-6, and TNF-α *in vivo* and *in vitro*. Another study showed that MG inhibited hepatic steatosis-induced NLRP3 inflammasome activation through the restoration of autophagy to promote Heme Oxygenase-1 (HO-1) signalling. HO-1 is an anti-inflammatory, antioxidant and neuroprotective inducible enzyme (Campbell et al., 2021), which is capable of ameliorating oxidative stress and inflammatory responses (Kuo et al., 2020).

#### 4.4.7 Salidroside (SAL)

SAL, a phenylpropanoid glycoside compound, is the major active ingredient of *Rhodiola rosea*. Earlier studies have shown that salidroside has pharmacological properties, including antioxidant and metabolic regulation, and exerts therapeutic effects on type 2 diabetes (Zheng et al., 2015; Wu et al., 2016). This study indicated that salidroside defends against NAFLD by alleviating hepatic lipid metabolism, reducing the release of inflammatory factors, and inhibiting NLRP3 inflammasome activation *in vivo* and *in vitro*, which were related to the regulation of the TXNIP/NLRP3 pathways (Zheng et al., 2018).

## 5 Conclusion and perspectives

We have summarized the latest research results on the improvement of NAFLD afforded by the regulation of NLRP3 by plant-derived active ingredients. First, we systematically discussed the mechanism of the NLRP3 inflammasome in the development of NAFLD, which mainly participates in the occurrence and development of NAFLD by mediating the inflammatory response and pyroptosis. We then described in detail the effects of these plant-derived compounds on the phenotype of the NAFLD model *in vitro* and *in vivo.* In addition, we summarized their effect on the NLRP3 inflammasome and its related indicators, procaspase-1, active caspase-1, ASC, pro-IL-1β, and IL-1β, and pyroptosis-related indicators, as well as the mechanism by which these compounds inhibit the NLRP3 inflammasome to improve NAFLD.

The NLRP3 inflammasome plays an important role in the development and progression of NAFLD. Excitingly, many plant-derived active ingredients can improve NAFLD by inhibiting NLRP3 inflammasome. Therefore, identifying NLRP3 inhibitors in plant-derived active compounds is of great significance for drug discovery as they are mostly safe, widely distributed, and available. These compounds included terpenoids, flavonoids, phenols, and alkaloids. Antcin A, a terpenoid, can improve NAFLD by binding to NLRP3 to inhibit both the inflammatory response and pyroptosis. Gardenoside, mangiferin, berberine, and dieckol can also inhibit inflammatory response and pyroptosis. We suspect that these five compounds may elicit a better anti-NAFLD effect in combination because of synergistic inhibitory effects. Glycyrrhizin inhibits NLRP3 initiation through the TLR/NLRP3 pathway, and *de novo* inhibition of NLRP3 may explain why glycyrrhizin exerts a strong anti-inflammatory effect. Naringin and antcin A have inhibitory effects on NLRP3 inflammasome assembly, which could provide a reference for the development of inhibitors of NLRP3 assembly. Most of these compounds ameliorated NAFLD by acting on NF-κB/NLRP3 and TXNIP/NLRP3 upstream signals or by combating oxidative stress by promoting NF-E2-related factor 2 (Nrf2). Nrf2 is an essential transcription factor that regulates an array of detoxifying and antioxidant defense gene expression in the liver (Beyer and Werner 2008). Berberine and ginsenoside have been studied the most. DMTHB, a derivative of berberine, has a significant effect on improving NAFLD models *in vitro* and *in vivo*. The 31 plant-derived compounds mentioned herein exhibit inhibitory effects on NLRP3, serving as a reference for the development of treatments for NAFLD.

Nevertheless, much work remains to be done to screen for natural drugs that are effective against NAFLD. Our analysis found that most of the current studies are limited to descriptive findings, and only a few studies have explored the potential interaction between these compounds and NLRP3, whereas most of the studies lack in-depth mechanistic research. For example, one included study of using resveratrol in treating NAFLD, only the effects of resveratrol on the expressions of NLRP3, caspase-1 and ASC proteins were observed, but the mechanism was not further explored. In addition, in a study on mangiferin inhibiting pyroptosis, the authors only showed the effect of mangiferin on cell pyrosis marker GSDMD, but did not observe the pathological manifestation of pyroptosis. Therefore, future studies on these mechanisms in depth and comprehensiveness will contribute to a better understanding of the complex mechanisms of NLRP3 inflammasome activation. All of the compounds mentioned herein inhibit NLRP3, but further research is needed to reveal whether this relationship is direct. Although the binding site of NLRP3 inflammasome-associated proteins to a natural product has been identified, further studies are needed to uncover the mechanism of action of potential NLRP3 inflammasome inhibitors and to screen for molecules that can selectively antagonize NLRP3 for NAFLD treatment. The large dose difference of compounds used in different studies may lead to off-target effects and false-positive results. Therefore, standardization of NLRP3 specific inhibitory dose is of great significance for the safety and efficacy of future NAFLD drug studies. Although many biological effects of these plant-derived active ingredients have been confirmed in cell and animal models in the treatment of NAFLD, insufficient clinical trials have been conducted to confirm the efficacy of these plant-derived substances for NAFLD, and further clinical trials are warranted.

In summary, we have discussed plant-derived active ingredients that can improve NASH by mediating NLRP3 to alleviate the inflammatory response or pyroptosis. In light of the collected data described herein, we also propose relevant suggestions and improvement measures, which are of significance for the development of NAFLD treatment drugs.

## Glossary

NAFLD: non-alcoholic fatty liver disease
NLRP3: pyrin domain-containing protein 3
IL-1β: interleukin (IL)-1β
NAFL: non-alcoholic fatty liver
NASH: non-alcoholic steatohepatitis
DAMPs: damage-related molecular patterns
PAMPs: pathogen-associated molecular patterns
KCs: Kupffer cells
FDA: Food and Drug Administration
TLR: toll-like receptor
NF-κB: nuclear factor kappa-B
ROS: reactive oxygen species
HSCs: hepatic stellate cells
ALT: alanine transaminase
AST: aspartate transaminase
BMDMs: bone marrow-derived macrophages
PA: palmitic acid
GSDMD: Gasdermin D
FFD: fast food diet
TC: total cholesterol
HFD: high-fat diet
TXNIP: thioredoxin interaction protein
LPS: Lipopolysaccharide
ATP: adenosine triphosphate
TG: triglyceride
TNF-α: tumor necrosis factor -α
FFA: free fatty-acids
CTCF: CCCTC-binding factor silencing
DPP4: dipeptidyl peptidase-4
MCD: methionine-choline deficient diet
XO: xanthine oxidase
HSP90: heat-shock protein 90
SGT1: solanidine galactosyltransferases
NAD+: nicotbambeadenbe dinuclotide
SIRT2: sirtuin type2
PINK1: PTEN-induced kinase 1
HO-1: Heme Oxygenase-1
